# Excitation and electroporation in genetically engineered excitable S-HEK cells exposed to electric pulses of different durations

**DOI:** 10.1038/s41598-025-06989-5

**Published:** 2025-07-02

**Authors:** Tina Batista Napotnik, Tina Cimperman, Lea Rems

**Affiliations:** Faculty of Electrical Engineering, University of Ljubljana, Tržaška Cesta 25, 1000 Ljubljana, Slovenia

**Keywords:** Electroporation, Excitable cells, Action potential, Pulse duration, Intracellular calcium, Cell biology, Medical research, Biophysics

## Abstract

Electroporation affects action potential generation in excitable cells such as nerve, muscle and cardiac cells. Since electroporation-based treatments use different pulse protocols, we investigated how electric pulses of different duration (from 500 ns to 1 ms) trigger action potentials and cause electroporation in genetically engineered cultured excitable cell model. Transmembrane voltage was monitored using a fluorescent potentiometric probe and fluorescence microscopy. We triggered similar responses in these cells using electric pulses of all pulse durations. At lower electric fields, we stimulated action potentials and at higher electric fields, electroporation occurred: the action potentials were gradually prolonged and eventually, ended up in sustained depolarization. For shorter pulses, higher electric fields had to be used to achieve the same effect. However, the shape of the action potential was affected by pulse duration. With shorter pulses (500 ns–10 µs), the action potentials were much longer already at the excitation thresholds, due to more prominent effect of electroporation, compared to longer pulses (e.g., 1 ms) where action potentials were triggered by classical electrostimulation (i.e., excitation) without electroporation. Moreover, we detected a complex, biphasic intracellular calcium response in excitable S-HEK cells that was absent in non-excitable NS-HEK version of these cells.

## Introduction

Excitable cells (such as neurons, muscle, and cardiac cells) have the ability to generate and conduct electrical signals called action potentials (AP) in response to membrane depolarization that activates voltage-gated ion channels. These ion channels are expressed in the plasma membrane of all electrically excitable cells and are responsible for generating and propagating the AP^1,2^.

If we expose the excitable cells to low intensity electric pulses (with pulse duration usually between a few tens of µs to tens of ms) we can depolarize these cells to reach the threshold for opening of voltage-gated sodium channels (or, in some cases, voltage-gated calcium channels) which initiates the AP^2,3^. This electrostimulation is used in many medical applications such as cardiac pacemakers, heart defibrillation and deep brain stimulation^4–6^. However, if we expose the cells to electric pulses of higher intensity, we also achieve electroporation. Cells’ plasma membrane becomes temporarily permeabilized for the molecules that are otherwise poorly permeant^7^. Electroporation is increasingly used in numerous applications in medicine^8–11^, biotechnology^12^ and food technology^13^.

The additional ionic currents through electroporated plasma membrane can affect cell excitability^3,14^. This interaction between excitability and electroporation is very important in electroporation-based medical treatments. Namely, electroporation can affect the occurrence, shape and propagation of AP^3,15–18^. The safety gap between excitation and electroporation is important for medical applications where cells need to be repeatedly stimulated for longer periods of time without any membrane damage^3^. However, with short pulses in a nanosecond regime (nsEP; under 1 µs) it was already shown that in cells in vitro, excitation was always accompanied by electroporation^15,19,20^. Since different electroporation-based therapies use electric pulses of different durations^21^, it is important to explore further the effects of different pulses on excitable cells.

In our previous study^22^, we investigated the excitation and electroporation triggered by single 100 µs electric pulses of increasing amplitude on a simple excitable cell model – genetically engineered human embryonic kidney (HEK) cells. These cells express a minimal complement of sodium and potassium ion channels (Na_V_1.5 and K_ir_2.1) required for excitability^23–25^. They are easy to grow and propagate and they form robust AP as monitored optically under a fluorescence microscope using a potentiometric probe^22^. With the use of an excitable S-HEK and a non-excitable NS-HEK variant of this genetically engineered cell line we established that electroporation appears already at the voltages close to the threshold for excitability when exposing the cells to 100 µs long pulses. Moreover, we demonstrated that electroporation leads to the prolongation of AP and, at higher pulse amplitudes, to sustained depolarization. The sustained depolarization can be explained by an additional non-selective current through the membrane due to electroporation. We also observed that 100 µs pulses caused an increase in intracellular Ca^2+^ close to the threshold for triggering AP. This calcium response was different in excitable S-HEK cells than in their non-excitable counterparts, NS-HEK cells, which express Na_v_1.5 channels only. In S-HEK cells, the first peak was followed by a second, amplified peak that was never observed in NS-HEK cells.

The aim of this study was to investigate the effects of pulses of different durations (500 ns to 1 ms) on excitability and electroporation in genetically engineered excitable S-HEK cells. We triggered AP in S-HEK cells with pulses of all durations. However, the AP characteristics changed with decreasing pulse duration, indicating that excitation is accompanied by electroporation for pulse duration of 10 µs and shorter. All the pulses also triggered a complex calcium response in S-HEK cells but not in NS-HEK cells. Our findings are relevant to the emerging new electroporation-based therapies on excitable tissues including the heart^8,26^, brain^27^ and muscles^28^. In these therapies, better understanding of short- and long-term effects of electroporation on excitability of targeted tissues is needed to guide interpretation of results and enable optimization of the protocols for more effective outcomes.

## Results

### Electric pulses of different durations (500 ns – 1 ms) cause field-dependent responses of transmembrane voltage in excitable S-HEK cells

**Fig. 1:**
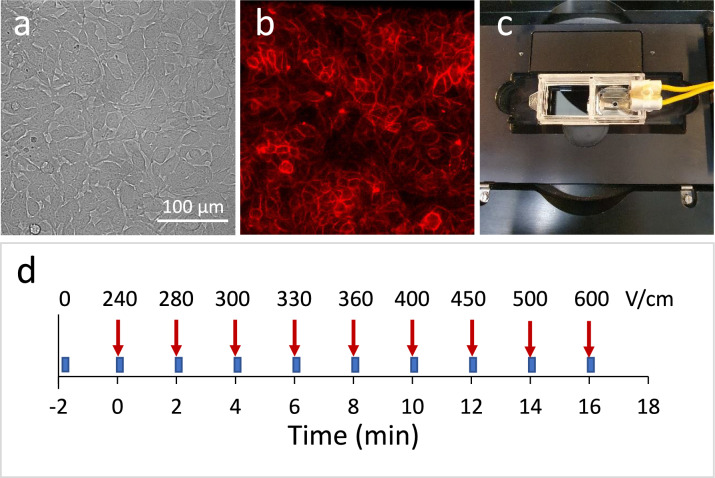
Experimental setup. Microscopic images of S-HEK cells, brightfield (**a**) and fluorescence (**b**) where cells are stained with a fluorescent potentiometric dye ElectroFluor630. Imaging chamber with inserted parallel wire electrodes under the microscope (**c**). Experiment timeline (**d**): time-lapse image acquisitions (blue tabs, each acquisition is 2.8 s long) and delivery of pulses of increasing electric field strength (red arrows, a pulse is applied at time 0.324 s of each image acquisition). The sequence of electric field strengths is shown for 10 µs pulses (for other pulse durations see Methods, Table 1).

S-HEK cells were grown as a monolayer within an imaging chamber and stained with fluorescent potentiometric dye ElectroFluor630 (Fig. 1a–c). They were exposed under the microscope (Fig. 1c) to a sequence of single electric pulses delivered 2 min apart. Each subsequent pulse had a higher amplitude (Fig. 1d). For each pulse delivery we captured 2.8-s-long time-lapse acquisitions with the pulse applied at the 10th acquired image. We tested pulses of four different durations (500 ns, 1 µs, 10 µs, and 1 ms). Results for 100 µs pulse duration were taken from our previous study^22^. For each pulse duration we adjusted the range of the pulse amplitudes applied within a sequence, since shorter pulses required significantly higher electric field strengths for triggering a response.

**Fig. 2:**
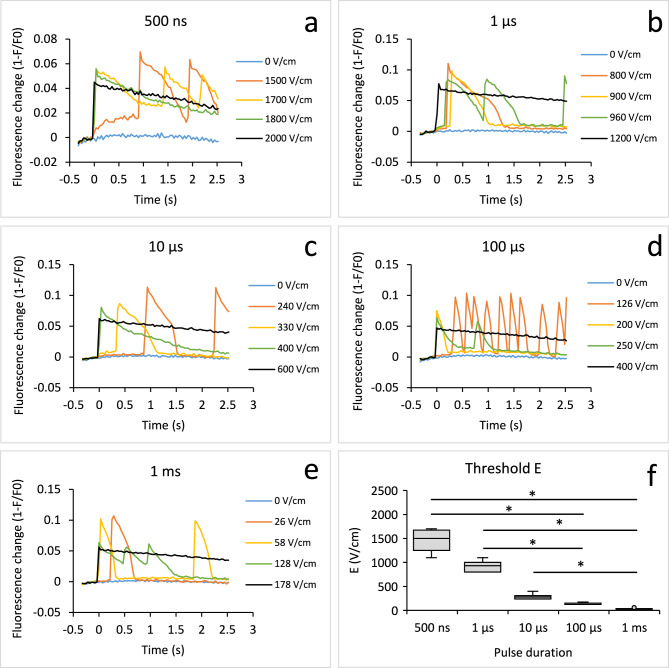
TMV responses to pulses of different durations in excitable S-HEK cells. Each panel (**a**-**e**) shows responses from a single representative sample, which was exposed to a sequence of pulses with increasing electric field E delivered 2 min apart to each sample of cells. Responses for selected E are shown; pulses were delivered at time zero. Presented curves show ElectroFluor630 fluorescence signal combined from the membranes of all the cells in the field of view and corrected for photobleaching. Note the different y axis ranges. (**f**): TMV response threshold E depending on the electric pulse duration (box-whiskers plot, *statistical difference between thresholds, ANOVA On Ranks followed by Dunn’s test for all pairwise multiple comparisons, p < 0.05). Number of experiments (N): 500 ns: 8, 1 µs: 10, 10 µs: 10, 100 µs: 13, 1 ms: 15.

From each time-lapse acquisition we extracted the relative change in fluorescence of all cell membranes in the field of view to obtain a time course of the collective change in transmembrane voltage (TMV) of these cells^29^. The signals obtained showed that we triggered qualitatively similar types of TMV responses in cells with all pulse durations (Fig. 2a–e). Below a certain threshold E, there was no response. Above the threshold, the cells responded with single or multiple action potentials (AP), seen as a rapid change (rise and fall) in TMV. For a typical single or multiple AP, see Fig. 2d, yellow and orange line, respectively. With increasing electric field (E), AP typically became longer (e.g., Fig. 2b, orange line). At the highest E used in experiment, TMV did not recover to the baseline in time of image acquisition (around 2.5 s after pulse application) and exhibited a sustained depolarization (black lines in all the Fig. 2a–e). Shorter pulses required much higher E to elicit a response. Median TMV response threshold E values (where AP first appeared in each sample of cells) were 1500, 930, 280, 126 and 38 V/cm for 500 ns, 1 µs, 10 µs, 100 µs and 1 ms, respectively (Fig. 2f).

We noticed that the responses at higher E (mostly sustained depolarization) were smaller than AP triggered by lower E. Complete membrane depolarization (~ 0 mV) should result in somewhat lower fluorescence change than an AP, since during an AP the TMV typically becomes positive, as evidenced through patch-clamp measurements in S-HEK cells^30^. The smaller response at higher E can also be partially attributed to the gradual translocation of the potentiometric dye from plasma membrane into the cells, which decreases the fluorescence signal at the plasma membrane and affects the sensitivity of the measurements (Supplementary information 1, Fig. S1)^31,32^. Moreover, this smaller response may partially be a consequence of cumulative effects of electroporation^33^. Nevertheless, we were still able to trigger AP in these cells after the entire sequence of pulses was completed, confirming that the cells were able to recover from sustained depolarization (see Supplementary information 1, Fig. S2).

**Fig. 3:**
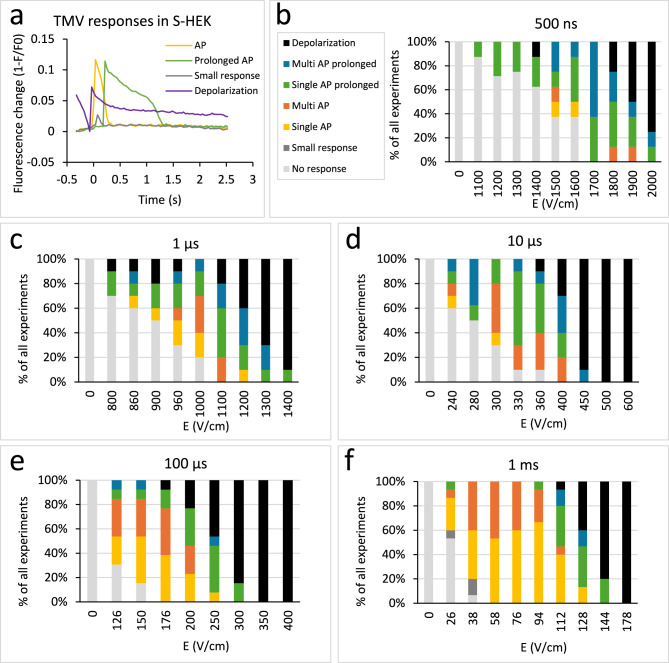
Different TMV responses in S-HEK after applying electric pulses of different durations and increasing electric field E. Representative figures of action potential (AP), prolonged AP, small response and depolarization after persisting AP from previous pulse application are shown (**a**). ElectroFluor630 fluorescence signal was combined from the membranes of all the cells in the field of view and corrected for photobleaching. (**b**-**f**): Different classes of TMV responses are shown after applying electric pulses of different pulse duration and increasing E, expressed in % of all experiments. The legend in (**b**) applies to all the panels (**b**-**f**). Number of experiments the same as in Fig. 2.

Samples that responded with multiple AP showed the highest number of peaks at lower and moderate E (above the threshold). The number of peaks diminished with increasing E, where most samples responded with sustained depolarization (see Supplementary information 1, Fig. S3). Note that while sustained depolarization typically resembled a step function, we counted the beginning of such step function as a “peak”. The highest average number of AP during image acquisition ranges from 1.7 after 1 ms pulse and 2.8 after 10 µs pulse application. In some cases (7 out of 57), the cells responded to electric pulses of moderate E with multiple AP that persisted for two minutes interval until the next pulse was applied. These samples exhibited a peak or high initial value before the pulse application during the next time-lapse acquisition (see Fig. 3a, purple line). These peaks were not counted towards the total number of peaks in Fig. S1.

To illustrate the change in response type with increasing E, we used a simple rule-based classification and divided the responses into seven distinct classes (Fig. 3): 1. No response, 2. Small response, 3. Single AP, 4. Multiple AP, 5. Prolonged AP, 6. Prolonged multiple AP, 7. Depolarization. The classification was based on the number of peaks, first peak amplitude, and the time it took for the signal to decrease by 50% (t_50_) and 75% (t_75_) of its first peak value (see Supplementary information 2 for details on classification). To define an AP, we analysed the threshold responses to 1 ms pulses, which are often used in classical electrostimulation^34,35^. All but one AP had t_50_ ≤ 450 ms and all had t_75_/t_50_ < 1.8. This condition was used to separate APs from prolonged APs (t_50_ > 450 ms or t_75_/t_50_ > 1.8). Depolarization was assigned to all signals that did not recover to 75% within the observation time. For 1 ms pulse there were 3 samples that showed a small peak at the threshold E followed by a typical AP response at subsequent E. Such small peak was classified as small response.

The results of this classification (Fig. 3) demonstrated that longer pulses (1 ms, 100 µs) mainly triggered single or multiple AP (see yellow and orange bars in Fig. 3e,f) at lower E values. With increasing E, AP became prolonged (green and blue), and the highest E values resulted in sustained depolarization (black). In contrast, the shortest pulses (500 ns), more often triggered a prolonged AP already at lower E while still resulting in sustained depolarization at the highest E (Fig. 3b). Classifications for intermediate pulse durations (1, 10 µs) were somewhere in between those for the longest and shortest pulses (Fig. 3c,d).

### Pulse duration affects the parameters of threshold TMV responses to electric pulses in S-HEK cells

To further explore the differences between threshold responses triggered by the longer and shorter applied pulses, we determined the full width at half maximum (FWHM) and the amplitude of the first peak that appeared in each sample at the threshold E, i.e., the first response of the sample. Similarly, we also determined the delay between the pulse application and time of the first peak in the first response.

**Fig. 4:**
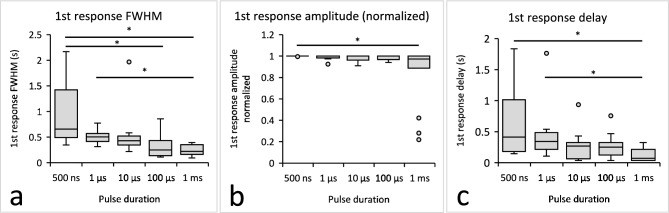
Analysis of the first TMV response of each S-HEK cell sample at the threshold electric field E. Full width at half maximum FWHM (**a**), normalized amplitude (**b**), and response delay (**c**) after applying electric pulses of different durations are shown. Box-whiskers plot, *statistical difference between values at different pulse durations, ANOVA On Ranks followed by Dunn’s test for all pairwise multiple comparisons, p < 0.05. Number of experiments the same as in Fig. 2.

The FWHM of the first responses was much larger after applying shorter pulses than longer (Fig. 4a, significant differences were 500 ns vs. 1 ms and 100 µs, and 1 µs vs. 1 ms). The median FWHM was 659 ms after the 500 ns pulse and 222 ms after the 1 ms pulse.

The amplitudes (peak height normalized to the maximal response of the sample) were the same for all pulse durations except after 1 ms pulse (Fig. 4b, statistically significant difference was 500 ns vs. 1 ms). This was a consequence of three cases of small responses (Fig. 3a, grey line) that we have noticed only after 1 ms pulse delivery (Fig. 3f).

Previously, we observed a delay between the 100 µs pulse application and TMV response that diminished with the increasing E^22^. A similar delay was detected in this study after applying the pulses of all durations (Fig. 4c, see also Supplementary information 1, Fig. S4). The first response delay was much longer after applying shorter pulses than longer pulses (Fig. 4c, statistically significant difference was 500 ns vs. 1 ms, and 1 µs vs. 1 ms). The median delay of the threshold TMV response was 414 ms after the 500 ns pulse and 72 ms after the 1 ms pulse.

### Strength-duration curve for excitation and electroporation

**Fig. 5:**
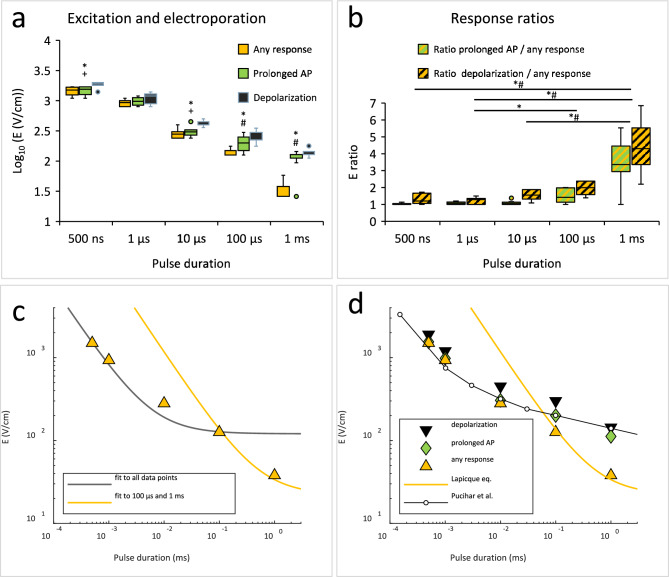
Excitation and electroporation in S-HEK cells after applying electric pulses of different durations. In (**a**), thresholds for any response, prolonged action potentials (AP) and sustained depolarization (depolarization), and in (**b**), response threshold ratios are shown in box-whiskers plots. In (**a**), statistical differences between thresholds are denoted for each pulse duration: * any response vs. depolarization, + prolonged AP vs. depolarization, and # any response vs. prolonged AP, ANOVA on Ranks followed by Dunn test for all pairwise multiple comparisons, p < 0.05. In (**b**), statistical differences between pulse durations are denoted for each ratio: * ratio depolarization threshold / any response threshold, and # ratio prolonged AP threshold / any response threshold, ANOVA on Ranks followed by Dunn test for all pairwise multiple comparisons, p < 0.05. Number of experiments the same as in Fig. 2. (**c**) Lapicque’s equation fit to the median threshold E for any response considering all data points (grey curve, *b* = 128 V/cm, *c* = 5.4 µs) and data points for 100 µs and 1 ms pulse only (yellow curve, *b* = 22 V/cm, *c* = 0.5 ms). (**d**) Graph showing median threshold values for any response, AP prolongation, and sustained depolarization, together with the fitted Lapicque’s curve and scaled curve from Pucihar et al.^41^, representing strength-duration relationship for excitation and electroporation, respectively. The curve fitting was performed in Matlab 2021b (Mathworks, USA) using the function fit().

Our previous computational modelling^22^ demonstrated that electroporation prolongs APs and leads to sustained depolarization due to increased membrane conductivity and associated ion leak currents. Thus, we considered that AP prolongation and sustained depolarization can be used as indicators of electroporation. For each sample we determined the lowest E at which any response, prolonged AP, and sustained depolarization occurred. Box plots showing these threshold E values for each pulse duration are presented in Fig. 5.

With longer pulses (1 ms and 100 µs), the thresholds for any response were significantly lower than for prolonged AP, whereby the later appeared close to the depolarization thresholds. This means that the first responses were AP (excitation in classical electrostimulation), and that electroporation (prolonged AP, sustained depolarization) occurred at E much higher than excitation thresholds. With shorter pulses (10 µs, 1 µs, 500 ns), the thresholds for any response and prolonged AP were not significantly different. This confirms that the APs were prolonged already at the excitation threshold (Fig. 5a), i.e., simultaneous excitation and electroporation.

The ratio between the electroporation threshold and the excitation threshold is often called a safety factor. The safety factor quantifies the safety of a electrostimulation protocol without adverse effects of electroporation^36^. The ratio between prolonged AP and any response threshold was much lower at shorter pulses (500 ns–10 µs) than at longer pulses (100 µs–1 ms). For example, at 500 ns pulse, the thresholds for any response and prolonged AP were the same (median ratio was 1), but at 1 ms pulse, the threshold E for prolonged AP was 3.4 times higher than the threshold E for any response. Similarly, the ratio between sustained depolarization and any response threshold was much lower at shorter pulses than at longer pulses. Median sustained depolarization threshold E was only 1.2 times higher than any response threshold E at 500 ns pulse and 4.3 times at 1 ms pulse (Fig. 5b). These results reaffirm that excitation and electroporation occur simultaneously when exposing the cells to shorter electric pulses and considerably more apart when exposing them to longer (1 ms and 100 µs) pulses.

The strength-duration curve for excitation generally follows the Lapicque’s equation: *E*_*thresh*_ = *b*(1 + *c*/*t*_*p*_)^37^, where *E*_*thresh*_ is the threshold stimulus, *b* is the rheobase, *c* is the chronaxie and *t*_*p*_ is the pulse duration. When we fitted this equation to the median threshold E values for any response across all pulse durations, the fit was poor (Fig. 5c, grey curve). However, restricting the fit to the 100 µs and 1 ms data — where our results suggest classical excitation — yielded a chronaxie of *c* = 0.5 ms, comparable to that reported for cardiomyocytes (0.5 − 3 ms^38–40^). We further compared the median thresholds for prolonged AP to data from Pucihar et al.^41^, who measured the electric field required to electroporate ~ 70% of CHO cells using pulse durations from 150 ns to 100 ms. Their reported electric fields were higher than ours, likely due to differences in cell arrangement: they electroporated isolated cells, whereas our cells were tightly packed and electrically connected. Using the least squares method, we scaled the electric field values from Pucihar et al. by a factor of *f* = 0.334 to align their curve with our data. The functional relationship between electric field and pulse duration observed by Pucihar et al. closely matched the relationship we observed for the median threshold E for AP prolongation and sustained depolarization (Fig. 5d).

### Calcium response

In our previous study using 100 µs pulses we observed that the pulses increased intracellular Ca^2+^ close to the threshold for triggering AP. This increase was characteristically different in excitable S-HEK cells than in their non-excitable counterparts, NS-HEK cells, which express Na_v_1.5 channels only. To study the effects of pulse duration on Ca^2+^, we exposed S-HEK and NS-HEK cells to the same pulse sequences of increasing E as in ElectroFluor630 experiments, monitoring intracellular Ca^2+^ using ratiometric imaging of the Fura-2 dye. Intracellular Ca^2+^ was monitored for 40 s, ~ 14 × longer than we monitored TMV, since the dynamics of calcium response was much slower than that of TMV.

**Fig. 6:**
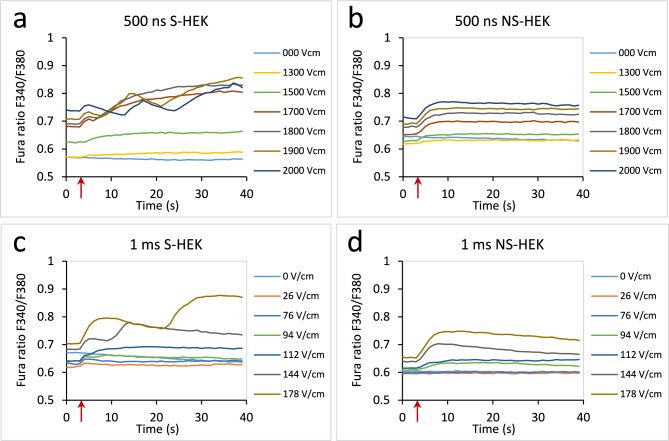
Ca^2+^ response in S-HEK and NS-HEK to the pulse sequence for different pulse durations in representative samples. (**a**) 500 ns, S-HEK cells, (**b**) 500 ns, NS-HEK cells, (**c**) 1 ms, S-HEK cells, (**d**) 1 ms, NS-HEK cells. Ca^2+^ responses were determined by Fura-2 ratio 340/380, averaged over the whole image. A pulse was delivered at around 4th second after the image acquisition started (red arrow).

We observed Ca^2+^ increase in both S-HEK and NS-HEK after all pulse durations (Fig. 6). Representative responses to sequences of 500 ns and 1 ms pulses are presented, similar responses were recorded also for 1 and 10 µs. However, two characteristic differences between S-HEK and NS-HEK can be immediately seen. While NS-HEK responded with a single transient Ca^2+^ increase (single peak, Figs. 6b,d), the response in S-HEK cells was more complex. After initial Ca^2+^ increase immediately after the pulse, often a second, much higher peak was observed (Fig. 6a,c). Sometimes, the higher peaks were even followed by additional peaks (up to 5, Fig. 6a). Due to this complex response, the increase in Ca^2+^ was typically higher in S-HEK than in NS-HEK cells.

**Fig. 7:**
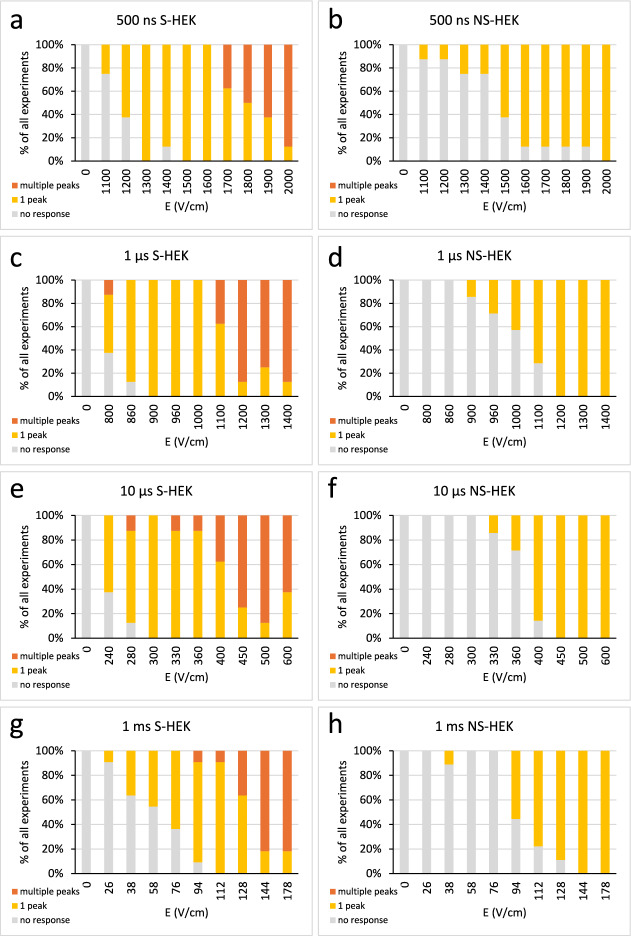
Classification based on the number of peaks in Ca^2+^ responses in S-HEK and NS-HEK. Number of experiments with 0, 1 or more Ca^2+^ peaks in S-HEK (**a**, **c**, **e**, **g**) and NS-HEK (**b**, **d**, **f**, **h**) after sequence of pulses of different durations (500 ns – **a**, **b**, 1 µs – **c**, **d**, 10 µs – **e**, **f**, 1 ms – **g**, **h**) are counted and expressed as % of all experiments. Number of experiments (N): 500 ns, S-HEK: 8, 500 ns, NS-HEK: 8, 1 µs, S-HEK: 8, 1 µs, NS-HEK: 7, 10 µs, S-HEK: 8, 10 µs, NS-HEK: 7, 1 ms, S-HEK: 11, 1 ms, NS-HEK: 9.

We quantified the number of peaks at each applied E in all experiments, and we classified the responses into those that showed no peaks, a single peak, and two or more peaks (Fig. 7). This analysis confirmed that, regardless of the pulse duration, multiple peaks were only observed in S-HEK cells. NS-HEK always responded with a single peak, no subsequent peaks were ever detected. Moreover, we noticed that in NS-HEK cells, Ca^2+^ responses occurred at higher E than in S-HEK cells for all pulse durations (significantly higher occurrence of responses at lower E in S-HEK than in NS-HEK, Fisher’s exact test).

**Fig. 8:**
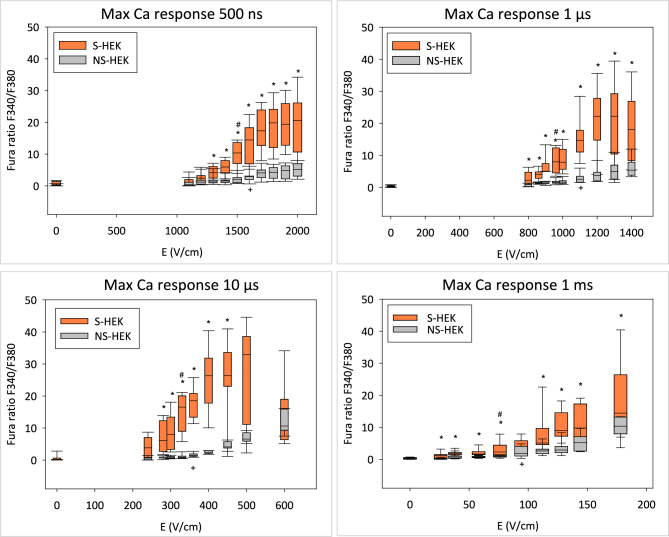
Maximum Ca^2+^ responses in S-HEK and NS-HEK cells after pulses of different durations (**a**: 500 ns, **b**: 1 µs, **c**: 10 µs, **d**: 100 µs, e: 1 ms). The maximum was determined as the maximum value of the Fura ratio in a given response minus the value at time t = 0 s before pulse application. Results are shown as box-whiskers plot, *statistical difference between S-HEK and NS-HEK cells (Mann–Whitney Rank Sum Test), # the lowest E where maximum Ca^2+^ response is significantly different from control in S-HEK cells, + the lowest E where maximum Ca^2+^ response is significantly different from control in NS-HEK cells (# and + ANOVA On Ranks, Multiple Comparisons versus Control Group – Dunn’s Method). Number of experiments the same as in Fig. 7. Note the different range of electric field strengths.

We further determined the maximum of Ca^2+^ response (Fig. 8). For all pulse durations this maximum was much higher in S-HEK cells than in NS-HEK cells. The response was higher in S-HEK cells already at lower E values where the second, amplified Ca^2+^ response in S-HEK cells was not yet observed (cf. Figures 7 and 8). We also determined the lowest E at which the maximum Ca^2+^ response is significantly different from control at 0 V/cm. In S-HEK, this E value was lower than in NS-HEK for all pulse durations (for S-HEK/NS-HEK; 500 ns: 1500/1600 V/cm, 1 µs: 960/1100, 10 µs: 330/360, and 1 ms: 76/94 V/cm). Similar was found when comparing the median threshold E for detectable Ca^2+^ response in S-HEK/NS-HEK (for 500 ns: 1200/1500 V/cm, for 1 µs: 800/1100, for 10 µs: 240/400, and for 1 ms: 58/94 V/cm).

It is interesting to compare the median threshold E for Ca^2+^ response in S-HEK cells with the median threshold for excitation, as determined from Fig. 2f. For 500 ns, 1 µs, and 10 µs the Ca^2+^ response occurred at lower E, whereas for 1 ms it occurred at higher E (Ca^2+^ response/excitation; for 500 ns: 1200/1500 V/cm, for 1 µs: 800/930, for 10 µs: 240/280, and for 1 ms: 58/38 V/cm).

## Discussion

### Triggering action potentials in S-HEK cells with electric pulses of different duration

Understanding how excitable cells respond to electric pulses of varying durations is fundamental for both electrostimulation applications and for elucidating the interaction between electroporation and excitability. With the use of a fluorescent potentiometric dye, we confirmed previously published results^15,19,42–44^ i.e., that we can trigger AP in excitable cells with pulses of different durations, ranging from 500 ns to 1 ms.

With decreasing pulse duration, much higher pulse amplitudes had to be used to trigger an AP. This is consistent with previous studies reporting strength-duration curves^39,42–44^. The median threshold electric field for triggering an AP with 1 ms pulse (30 V/cm) obtained in our study was higher than reported for isolated adult guinea pig ventricular myocytes (~ 5 V/cm^39^) and monolayers of neonatal rat ventricular myocytes (~ 4 V/cm^38^). This is expected, since cardiomyocytes are considerably larger (110–200 μm long axis length^39^) than S-HEK cells (~ 20 μm diameter). These thresholds in vitro are lower than reported in the paper from Semenov et al.^44^ for isolated mammalian cardiomyocytes (~ 70–90 V/cm). However, this study used electrodes smaller than the cardiomyocyte dimension and reported the highest calculated electric field strength in between the electrodes. They also observed bubble formation during 2 ms pulse application, which could effectively reduce the electric field strength to which the cells are exposed. The median threshold electric fields for triggering an AP in S-HEK cells with 500 ns and 1 µs pulse (1500 V/cm and 930 V/cm) are nevertheless comparable to that reported in Semenov et al. for a 800 ns pulse (~ 700–1300 V/cm), but higher than in isolated frog skeletal muscle or nerve, where the estimated threshold electric field strength inside the tissue required for excitation by 1 μs pulse was on the order of 100 V/cm^42,43^. Note that nerves and skeletal muscle cells are considerably longer/larger than S-HEK cells and cardiomyocytes. These comparisons validate our experimental model and suggest the role of cell size in determining excitation thresholds.

Regardless of pulse duration however, a single electric pulse triggered single or multiple AP. Multiple APs could be a result of propagating AP waves initiated by the applied pulse^30,45^, since HEK cells endogenously express gap junctions and make electrical connections when grown in a monolayer. Alternatively, additional APs were observed also in experiments on isolated murine ventricular myocytes and were attributed to L-type calcium channels^15^. Some voltage-gated calcium channels are indeed endogenously expressed in HEK cells^46^. Therefore, the exact mechanism of multiple AP response requires further investigation.

### Action potentials triggered by shorter pulses are different than those by longer pulses at the threshold electric field strength

After establishing that we could trigger APs with pulses of different durations, we examined the characteristics of these APs and found notable differences between those triggered by short versus long pulses at their respective threshold electric fields.

Pulses of 1 ms duration, the longest in our study, are often used for classical electrostimulation^34,35^. The AP that we recorded in S-HEK cells at the threshold electric field with 1 ms pulses ranged from 100 ms to a few hundreds of ms. AP shape and duration vary in different types of excitable cells and range from a few milliseconds in neurons and skeletal muscle cells to hundreds of milliseconds in cardiomyocytes. This is because the ion channel expression profile as well as ion channel characteristics differ between neurons and muscle cells^47^. S-HEK cells are genetically engineered to express cardiac voltage-gated sodium channels Na_V_1.5 and inward rectifying potassium K_ir_2.1 channels. K_ir_2.1 are not voltage-gated and are primarily responsible for controlling the resting voltage in excitable cells. Since they conduct only a low current during depolarization^48–50^, S-HEK cells require more time to repolarize than, e.g., neurons. The expression level of K_ir_2.1 channels varies somewhat between samples, contributing to the observed variability in AP duration^30,51^.

Despite this inherent variability, our results clearly demonstrated that APs become progressively longer as pulse duration decreases. The median AP duration (FWHM) triggered at threshold with 500 ns and 1 µs pulse was significantly longer than in AP triggered by 1 ms pulse (659 ms and 505 ms vs 222 ms). This prolongation with shorter pulses likely results from electroporation occurring simultaneously with excitation at the threshold level. Electroporation results in leak ionic currents across the membrane that contribute to AP prolongation, as demonstrated through computational modelling in our previous publication^22^. Since we collected the fluorescence signal from the whole field of view, AP properties could be to some extent affected by uneven responses in individual cells within the group due to the variations in size, shape, orientation, and ion channel expression level^52^. However, considering the fact that the S-HEK cells in the monolayer have quite similar size and a roughly round shape (Fig. 1b), are electrically coupled, and that AP propagates across the monolayer with high speed (compared to our image acquisition)^22^, this is unlikely. Moreover, the cell size and shape do not affect the extent of electroporation due to pulses in a nanosecond range (100 and 600 ns) as much as they do after longer, micro- or millisecond pulses^53,54^.

We also observed that the delay between pulse application and AP occurrence progressively increased as pulse duration decreased. This delay was significantly different for the shortest (500 ns, 1 μs) and the longest (1 ms) pulses. The delay observed with longer pulses is most likely related to the time it takes for the AP to propagate ~ 2.5 mm from the electrodes (where the electric field strength is locally the highest and thus AP is triggered first) to the middle between the electrodes, where the cells are monitored^22^. Considering the AP propagation velocity of roughly 34 mm/s (estimated in our previous study^22^), the propagation of AP from the electrodes to the middle position can result in the delay in the range of several tens of ms, accounting for a considerable fraction of the experimentally determined delay. However, the delay, which we observed with the shortest pulses was considerably longer and even exceeded 1 s when exposing the cells to 500 ns and 1 us pulses. This long delay could be explained by two potential mechanisms. First, shorter pulses may activate voltage-gated sodium channels after rather than during the pulse application. The activation could be due to electroporation-induced leak currents which take time to depolarize the cell to the threshold for ion channel activation^3,18^. Longer pulses, in contrast, have a higher probability of activating sodium channels during the pulse itself, resulting in shorter delays. Similar delayed excitation was observed in cardiomyocytes exposed to 200 ns pulses^15^. Second, shorter pulses with considerably higher amplitudes than longer pulses may cause a gap junction block and slow down or prevent AP propagation from the electrodes. It was shown previously that nsEP cause amplitude-dependent gap junction inhibition^55^. Further experiments with spatio-temporal analysis of AP propagation would be needed to confirm this mechanism.

Our results agree with other reports that excitation triggered by nanosecond electric pulses (nsEP; pulses in a sub-microsecond regime) was always accompanied by electroporation^15,19,20^. Moreover, our results indicate that electroporation occurs simultaneously with excitation even with 1 μs and to some extent with 10 μs pulses (see below), meaning that this phenomenon is not limited to nsEP. However, our results appear to contradict reports where thousands of AP were triggered by nsEP on nerve, muscle or perfused heart preparations without apparent fatigue or damage^56–59^. This discrepancy between isolated cells in vitro and tissues/ organs remains unresolved. It was suggested that the damage due to electroporation occurs at a small, undetectable subset of cells in the tissue near the electrodes^60^.

### Gap between thresholds for excitation and electroporation decreases with decreasing pulse duration

As we increased the electric field strength beyond the threshold E that triggered AP, we observed consistent progression in TMV responses across all pulse durations. AP typically became longer until the TMV reached a state of sustained depolarization that persisted for at least 2.5 s after pulse application (time of image acquisition). Our previous research established that both AP prolongation and sustained depolarization are clear indicators of electroporation^22^. We determined the threshold electric field strengths for any response, AP prolongation and sustained depolarization to obtain strength-duration curves for both excitation and electroporation.

When analysing the excitation threshold data with Lapicque’s equation, we found that only the data for longer pulses (100 μs and 1 ms) yielded a reasonable fit, producing a chronaxie of *c* = 0.5 ms, comparable to that reported for monolayers of neonatal rat ventricular myocytes (*c* ≈ 3 ms)^38^, aggregates of cardiac cells from chic embryos (*c* ≈ 1.6 ms)^40^, and isolated adult guinea pig ventricular myocytes (*c* ≈ 0.5–2 ms, depending on the cell orientation)^39^. Fitting the data for all pulse durations resulted in a poor fit and an unrealistic chronaxie of 5.4 μs. In contrast, the strength-duration relationship for AP prolongation and depolarization across our entire pulse range closely matched the functional relationship between pulse duration and electric field strength for electroporation as determined by Pucihar et al.^41^ based on monitoring Ca^2+^ uptake in CHO cells. This correlation substantiates our interpretation that AP prolongation and sustained depolarization indicate electroporation. Our curve-fitting analysis suggested that the threshold for classical electrostimulation exceeds the threshold for electroporation around pulse duration of ~ 60 µs. This implies that for pulses shorter than 60 µs, excitation in S-HEK cells is expected to be accompanied by at least some degree of electroporation. Similarly, a study on isolated cardiomyocytes showed considerably lower threshold electric fields for excitation than predicted by Lapicque’s curve for pulses ≤ 100 µs and suggested electroporation as a potential mechanism^39^.

The finding that longer pulses (100 µs, 1 ms) triggered AP (excitation), whereas shorter pulses (500 ns, 1 μs, 10 μs) resulted in AP generation accompanied by electroporation is supported by several lines of evidence. With longer pulses there was a significant gap between excitation and AP prolongation thresholds. Additionally, the median threshold electric field strengths for detectable calcium response (attributed mainly to uptake of calcium from the medium into cells through electroporated membrane, see next subsection) were higher than those for excitation, further confirming the separation between excitation and electroporation phenomena. In contrast, there was virtually no gap between thresholds for excitation and AP prolongation with shorter pulses, and the median threshold for calcium response was lower than for excitation. The shorter the pulses, the more profound were the effects of electroporation. For the shortest pulses (500 ns and 1 µs), the AP recorded at threshold electric field strength were significantly longer and delayed compared to 1 ms pulse. For 10 µs pulses, this difference in AP characteristics was not significantly different from 1 ms pulse—this intermediate behaviour suggests that electroporation begins to occur with excitation for pulses with duration of around 10 µs, which is shorter but close to the ~ 60 µs predicted by curve fitting.

Previous studies proposed that excitation evoked by pulses shorter than the fastest activation time of voltage-gated sodium channels (estimated between 11  and  20 μs^56^) occurs through a mechanism different from classical electrostimulation. Namely, sodium channels become activated and trigger an AP due to membrane depolarization caused by electroporation-induced leak currents, rather than the depolarization that is induced directly by the electric field during the pulse exposure^3,18,20^. Our results fully support this hypothesis; however, we cannot determine conclusively whether excitation with pulses ≤ 10 μs was caused by electroporation or whether excitation and electroporation simply occurred simultaneously due to threshold proximity.

The distinction between excitation mechanisms with different pulse durations has important implications for the safety gap between excitation and electroporation thresholds. The longer the electric pulses, the bigger is this gap. This aligns with reports from Gudvangen^61^ that combined thresholds of in vitro experimental results on cells (cell killing—irreversible electroporation) and isolated nerves (stimulation), and Mercadal^62^ that combined thresholds of in vitro experimental results for irreversible electroporation and simulated stimulation, albeit not for monopolar but for bipolar pulses. For applications where electric pulses are used for electrostimulation of excitable tissues (brain, muscles, nerves, heart) for long periods of time, a large gap between excitation and electroporation is favourable to avoid damage. Conversely, this large gap may pose challenges in electroporation-based applications that target excitable tissues, such as cardiac ablation with irreversible electroporation for treatment of arrhythmias^8^, ablation of brain tumours or epileptic zones^27^, and electroporation-mediated delivery of nucleic acids into skeletal or cardiac muscle for gene therapy^26^. As electric field strength decreases with distance from electrodes, it creates regions of irreversible electroporation (IRE), reversible electroporation and electrostimulation around the treatment site^62,63^. The smaller the gap between electroporation and excitation thresholds, the smaller will be the electrostimulated region. In the heart, this may help avoid electroporation-induced arrhythmias, which have been observed in defibrillation studies using millisecond pulses^64^. For instance, ablation of porcine cardiac muscle using 300 ns pulses caused no ventricular fibrillation or other arrhythmias, even though pulse delivery was not synchronized with the electrocardiogram^65^. Whether the altered shape of AP in the electroporated region or perturbation of gap junctions^55^ contributes to the absence of arrhythmias, remains to be investigated. Since bipolar pulses appear to be less arrhythmogenic than monopolar pulses^66^, it would be interesting to assess the gap between electroporation and excitation for monopolar and bipolar pulses of the same durations.

Along similar lines, it has been shown through computational simulations that by reducing the pulse duration, the same IRE ablation area can be achieved while reducing the area in which nerves are stimulated^61,62^. This explains why bipolar pulses of ~ 1 μs duration^63,67,68^, as well as shorter 100 ns pulses^69^, reduce pain and muscle contraction compared with longer 100 μs pulses in electroporation-based applications such as tumour treatment. However, reduction of the electrostimulated region in such cases is not necessarily a consequence of the reduced gap between electroporation and excitation thresholds in one cell type (as shown in our study), but can be due to the different geometries of treated cells (e.g., round tumour cells vs. elongated neurons) and their different location relative to the electrodes^62^.

### Complex calcium response in S-HEK cells

We triggered intracellular calcium response with pulses of all durations, both in S-HEK and in NS-HEK cells. The pulses caused an increase in intracellular calcium, which peaked a few seconds to a few tens of seconds post pulse and slowly returned towards the baseline. As we have already seen with 100 µs pulses^22^, we have observed that with all pulse durations, the thresholds for calcium response were slightly higher in NS-HEK than in S-HEK cells and the differences were significant. This could be due to (1) differences in expression of endogenous voltage-gated calcium channels, that enable additional uptake of Ca^2+^ into the S-HEK cells, (2) detection and thresholding – we applied the same algorithm for Ca^2+^ peak detection in both S-HEK and NS-HEK cells, but due to overall lower uptake of Ca^2+^ in NS-HEK cells, their peaks were more difficult to detect at lower E, and/or (3) differences in electroporation thresholds due to different membrane composition.

Moreover, we have observed a complex, biphasic intracellular calcium response in S-HEK cells after the application of the pulses of all durations used (500 ns–1 ms), as was already reported in our previous study using 100 µs pulses^22^. After initial rise immediately after pulse application we detected a second calcium peak that was usually much higher than the first one. In some cases, there were even multiple peaks (up to 5 within 40 s). This complex calcium response occurred seconds or tens of seconds after the pulse application.

The first calcium peak is likely to be caused by electroporation of the lipid bilayer, and/or opening the endogenous voltage-gated calcium channels or other channels^22^. However, as we described before^22^, the second calcium peak may be attributed to an active amplification of calcium signal by more complex calcium pathways, such as calcium release from internal stores (ER) via ryanodine or inositol 1,4,5-trisphosphate (IP_3_) calcium-induced calcium release (CICR) receptors^70^, store-operated calcium entry^71^, intercellular calcium waves^72^, or depolarization-dependent influx of Ca^2+^ through voltage-gated calcium channels^73^. It was shown previously that nsEP triggered a release of Ca^2+^ from endoplasmic reticulum (ER) via IP_3_ receptors either by phosphatidylinositol 4,5-bisphosphate (PIP_2_) hydrolysis pathway^74–76^ or calcium-induced^77^. Semenov detected a biphasic calcium response (similar to our results) with nsEP that was attributed to the amplification of nsEP effects (uptake from permeabilized plasma membrane and/or efflux from ER) by CICR^77^. On the other hand, it was also proposed that nsEP recruit (store-operated) capacitive calcium entry from outside^78^, which may also lead to a biphasic calcium response. Depletion of the ER Ca^2+^ with thapsigargin can trigger capacitive store-operated Ca^2+^ entry from the outside^77^. In excitable chromaffin cells, nsEP elicited influx of Ca^2+^ through voltage-gated calcium channels (VGCC) in the plasma membrane^73,79^. The opening of VGCC by nsEP was dependent of depolarization and the influx of Na^+^, possibly through nanopores^73^. In cardiac myocytes, nsEP induced influx of Ca^2+^ through nanopores in plasma membrane, Ca^2+^ release from the internal stores, local Ca^2+^ transients and intracellular Ca^2+^ waves^20^. It should be however stressed that we observed the same biphasic response for all pulse durations, not just for nsEP.

Our current data cannot decipher the mechanism behind the second Ca^2+^ peak in S-HEK cells and explain why it occurs only in excitable S-HEK cells but not in NS-HEK – non-excitable version of the same cells that in theory, differ only in expression of K_ir_2.1 channels. An in-depth study of these mechanisms is currently being carried out to elucidate calcium responses in these cells.

## Conclusions

By determining both excitation and electroporation thresholds on a genetically modified excitable cell model S-HEK cells after exposing them to pulses of different duration (500 ns–1 ms) we confirmed that electroporation has a significantly larger impact on cells already in an excitation range when using short pulses (i.e., 500 ns–10 µs). This leads to prolonged action potentials at excitation thresholds with shorter pulses. This was not observed only with the use of nsEP (i.e., pulses in the ns regime, under 1 µs), but also with pulses up to 10 µs. Moreover, the safety factor between electroporation and excitation is much lower when using shorter pulses than longer pulses (e.g., 1 ms). The interplay between excitation and electroporation must be considered in electroporation-based therapies and treatments when excitable cells are affected. In this way, the optimization of treatment pulse protocol can be facilitated.

## Materials and methods

### Cells

For experiments, an excitable, genetically engineered HEK cell line was used, developed and generously provided by the group of Adam E. Cohen at Harvard University^24,25^. These cells have since become available from ATCC (cat. no. CRL-3479). The cell line was derived from HEK-293T cell line (ATCC CRL-3216) and was genetically modified to constitutively express Na_V_1.5 channels together with a tet-on system of doxycycline-induced expression of K_ir_2.1 channels. By adding doxycycline to the culture medium, we obtain “spiking” S-HEK cells – cells with both Na_V_1.5 and K_ir_2.1 channels that can generate APs (“spikes”). Without doxycycline, cells with only Na_V_1.5 channels served as complementary non-excitable (“non-spiking”) NS-HEK cells.

The cell line was propagated from week to week as NS-HEK cells, in Dulbecco′s Modified Eagle′s Medium high glucose growth medium (D5671, Sigma-Aldrich/Merck KGaA, Darmstadt, Germany), supplemented with 10% fetal bovine serum (F2442, Sigma-Aldrich/Merck), 2 mM glutamine (G7513, Sigma-Aldrich/Merck), penicillin–streptomycin (P07681, Sigma-Aldrich/Merck; penicillin 100 U/ml, streptomycin 100 µg/ml), 2 µg/ml puromycin (Thermo Fisher Scientific Inc., Waltham, Massachusetts, U.S.), 5 µg/ml blasticidin (Thermo Fisher Scientific), and 200 µg/ml geneticin (Thermo Fisher Scientific).

Two or three days prior to experiments, the cells were seeded to poly-L-lysine-coated 2-well Nunc Lab-Tek II Chambered #1.5 German Coverglass System (155379, Thermo Fisher Scientific) at a concentration 2.5 × 10^5^ or 10^5^ cells seeded per well, respectively. For generation of S-HEK cells, 4 µg/ml doxycycline (D9891, Sigma-Aldrich/Merck) was added to cells when seeded for a doxycycline-induced expression of K_ir_2.1 channels. NS-HEK cells were grown in Lab-Tek chambers in culture medium without doxycycline. Only passages lower than 15 were used in the experiments.

### Monitoring changes in transmembrane voltage

For monitoring changes in transmembrane voltage, including APs, we used ElectroFluor630 (Di-4-ANEQ(F)PTEA, Potentiometric Probes, Farmington, CT, USA) as described in our previous papers^22,29^. Briefly, cells were labelled with 12 µM ElectroFluor630 dye in culture medium for 20 min in a refrigerator (4 °C), washed with Tyrode solution (2 mM KCl, 125 mM NaCl, 2 mM CaCl_2_, 1 mM MgCl_2_, 10 mM HEPES, 30 mM glucose, pH 7.3) and finally, a low K^+^ Tyrode solution (0.5 mM KCl, 126.5 mM NaCl, 2 mM CaCl_2_, 1 mM MgCl_2_, 10 mM HEPES, 30 mM glucose, pH 7.3) was added to cells for more robust triggering of APs. Experiments were performed at room temperature.

As in our previous work^22^, the cells were exposed to a sequence of pulses of increasing voltage delivered 2 min apart, only this time we performed experiments using pulses of five different durations: 500 ns, 1 µs, 10 µs, 100 µs and 1 ms. The results for 100 µs pulses were obtained already in the previous study^22^ and were added to the present study. The electric fields (estimated as the applied voltage-to-electrode distance ratio) of the consecutive pulses for each pulse duration are presented in Table 1, next to the exposure time points. 2 min interval between pulse exposures was set as a compromise between minimizing the possible cumulative effects of consecutive pulses and minimizing the duration of an experiment to prevent deterioration of labelled cells under the microscope.

**Table 1:** The electric fields (estimated as the applied voltage-to-electrode distance ratio) of the consecutive pulses for each pulse duration, next to the exposure time points.

Pulse delivery time	E (V/cm)				
	500 ns	1 µs	10 µs	100 µs	1 ms
0 min	0	0	0	0	0
2 min	1100	800	240	126	26
4 min	1200	860	280	150	38
6 min	1300	900	300	176	58
8 min	1400	960	330	200	76
10 min	1500	1000	360	250	94
12 min	1600	1100	400	300	112
14 min	1700	1200	450	350	128
16 min	1800	1300	500	400	144
18 min	1900	1400	600		178
20 min	2000				

Pulses were delivered using a prototype pulse generator^80,81^ and parallel Pt/Ir wire electrodes, with 0.8 mm diameter and 5 mm distance between inner edges, placed at the bottom of the Lab-Tek chamber. Following recommendations^82^, voltage and current were routinely monitored with a WavePro 7300A oscilloscope, a differential voltage probe ADP305 and a current probe AP015, all from Teledyne LeCroy, Chestnut Ridge, NY, USA. The highest electric current flowing through the sample during experiments was 9.3 A (500 ns pulse, 2000 V/cm), which corresponds to the power of 9.3 kW. A “worst case” scenario of temperature rise in the medium between the electrodes without any heat dissipation^83^ was 0.1 K (1 ms pulse, 180 V/cm). A two-minute delay between the applied pulses allowed the dissipation of the generated heat into the environment.

For monitoring AP and changes in TMV, cells were exposed to electric pulses under the microscope Thunder Imager Live Cell system for fluorescence microscopy with DMi8 inverted fluorescent microscope, deep-cooled 4.2 MP sCMOS Leica DFC9000 Gt fast camera, a system of 8 LED diodes (LED8), and a UV light source (CoolLED pE340fura) (all from Leica Microsystems GmbH, Wetzlar, Germany). Cells were observed with a 40 × objective and illuminated with excitation wavelength of 635 nm (50% LED power, exposure time of 10 ms, 4 × 4 binning). The emission was detected at around 700 nm wavelength using a DFT51010 filter set. Images in time-lapse mode were acquired using LAS X software (Leica Microsystems): one image every 36 ms, 80 images, 2.8 s total duration of image acquisition, the pulse was delivered at 10th image (at around 324 ms, triggered by the TTL signal of the microscope triggering system). A time-lapse without pulse exposure recorded at the beginning of each experiment served as a control. Field of view was always chosen in the area in the middle between the electrodes.

Image analysis was done in Matlab (Mathworks, Natick, MA, USA) to extract relevant parameters, as previously described^22^. Briefly, images (see raw signal data curves in Supplementary information 1, Fig. S1) were thresholded to extract the time-dependent fluorescence signal from membrane regions in the entire captured image to improve the signal-to noise ratio. The reported signals thus show fluorescence change from all cell membranes in the image, not from single cells. The extracted signals were also corrected for photobleaching and translated on y-axis such that the minimum value of each signal representing the relative fluorescence change (1 – F/F_0_) was equal to 0. Signals were then processed using a custom Matlab algorithm to determine the parameters of the signal: number of peaks (*N*), maximum response, amplitude of the first peak (*A*), delay between pulse application and time of the first peak (t_delay_), and time from the first peak to 25, 50, 75, and 90% recovery (t_25_, t_50_, t_75_, t_90_). Sometimes the predicting pulse evoked AP oscillations that persisted until the application of a new pulse. In such cases, any peak that was identified before pulse application was removed from the analysis. Graphs of all signals, annotated with the extracted parameters, were visually inspected to verify the correctness of the determined parameters.

In a few cases, S-HEK cells did not generate AP due to too low or too high cell culture density or exhibited spontaneous AP^33^. These samples were eliminated from the study. Also, for 100 μs pulses we took only the results from S-HEK samples that triggered an AP in our previous study^22^.

### Monitoring intracellular calcium

Intracellular Ca^2+^ was monitored in ratiometric measurements with fluorescence microscopy using a fluorescent Ca^2+^ indicator Fura-2 acetoxymethyl (AM) ester (F1221, ThermoFisher Scientific)^22,84,85^. Cells in Lab-Tek chambers were labelled with 2 µM Fura-2 AM in culture medium at 37 °C for 30 min, 3 × washed with Tyrode solution and transferred to low K^+^ Tyrode solution which contained 2 mM CaCl_2_.

Cells were then exposed to the same pulse sequences as in ElectroFluor630 experiments. Pulses were delivered using the same electrodes, pulse generator, oscilloscope and voltage and current probes as in ElectroFluor630 experiments.

Cells were observed under Leica Thunder Imager Live Cell system with 63 × objective. They were illuminated with UV light source (CoolLED pE340fura, Leica) at two excitation wavelengths (340 nm and 380 nm). For 340 nm excitation, exposure time was 200 ms, 2 × 2 binning, Fluorescence Intensity Manager (FIM) 100%, and for 380 nm excitation, exposure time was 25 ms, 2 × 2 binning, FIM 17%. The emission was detected at 510 nm wavelength using Leica Fura LED filter set. Images were acquired with the software LAS X (Leica) in time-lapse acquisition mode: one pair of images (340 and 380 nm excitation) every second, 40 pairs of images, 39 s total duration of image acquisition, the pulse was delivered at 5th image (at around 4 s, triggered by the TTL signal of the microscope triggering system). A time-lapse without pulse exposure recorded at the beginning of each experiment served as a control.

For each time-lapse, the ratio images Fura-2 340/380 were calculated in ImageJ Fiji using the embedded function imageCalculator after subtracting a fixed background value (100) from both the images captured at 340 nm and 380 nm. Afterwards, we computed the average pixel intensity of each image in the time lapse to obtain the time-dependent signals R_340_/_380_(*t*). The signals R_340_/_380_(*t*) were then further analysed by a custom Matlab algorithm. Each signal was subtracted by its initial value, such that the signal value was 0 at time 0 s. The location of peaks in each signal was first identified using Matlab’s built-in function findpeaks using a defined threshold prominence and a minimum distance between peaks. If the maximum value of a signal was not automatically detected as a peak, it was manually added to ensure that major response peaks were captured. To further ensure robust peak detection, left prominences—the height difference between each peak and the lowest point on the path to its previous peak or beginning of the signal, were computed. Logical conditions were applied to filter valid peaks based on their amplitude and left prominence. The detected peaks in each signal were visually inspected and manually corrected in rare cases, where the algorithm misidentified the peaks.

### Statistical analysis

Statistical analysis was performed by using Excel (Microsoft, Redmond, WA, USA) and SigmaPlot 11.0 (Systat Software, Chicago, IL, USA). The results in figures are expressed as medians with bars Q1 and Q3. Normality of the data distribution was tested with the Shapiro–Wilk test. Since the normality assumption was not fulfilled in most cases, the nonparametric tests were used to assess the statistical significance of the differences. Significant differences (p < 0.05) in responses (TMV, Ca^2+^) were determined by Kruskal–Wallis One Way ANOVA on Ranks, followed by All Pairwise Multiple Comparison Procedures (Dunn’s Method).

## Supplementary Information

Supplementary Information 1.

Supplementary Information 2.

## Data Availability

The data presented in this study is available on request from the corresponding author.
