# Supplementary material for: Excitation and electroporation in genetically engineered excitable S-HEK cells exposed to electric pulses of different durations
Source: Sci Rep. 2025 Jul 2;15:23451. doi: 10.1038/s41598-025-06989-5 (PMC12223188; doi:10.1038/s41598-025-06989-5)
Supplement: Supplementary file 1 — Supplementary Information 1. [file 41598_2025_6989_MOESM1_ESM.pdf]

Supplementary information 1

Excitation and electroporation in genetically engineered excitable S-HEK cells exposed to electric pulses of different durations

Tina Batista Napotnik, Tina Cimperman, Lea Rems

University of Ljubljana, Faculty of Electrical Engineering, Tržaška cesta 25, 1000 Ljubljana, Slovenia

Supplementary figures

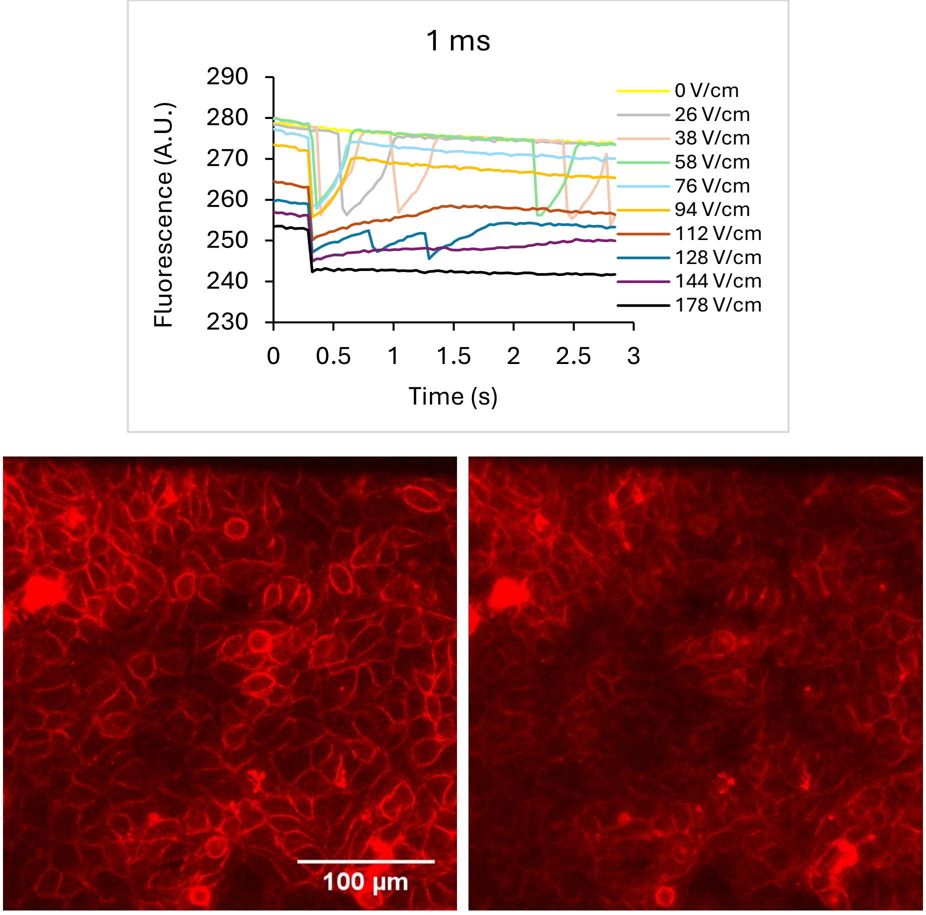

Figure S1: ElectroFluor630 (Di-4-ANEQ(F)PTEA) fluorescence signal from the whole field of view – raw data. S-HEK cells were exposed to a sequence of 1 ms electric pulses with increasing electric field (from 26 V/cm to 178 V/cm) every 2 min. Each curve represents a fluorescence signal during 2.8 s image acquisition when pulse was delivered. A pulse was delivered at around 324 ms of image acquisition, triggered by the TTL signal of the microscope triggering system. Note the gradual decrease in the baseline fluorescence signal along the applied pulse sequence. This decrease can be attributed to the gradual loss of plasma membrane fluorescence due to dye internalization into the cell and/or outflow into the extracellular solution, as seen in the fluorescence images. The left and right fluorescence images show ElectroFluor630 signal captured before pulse application (0 V/cm) and when applying the last pulse in the pulse sequence (178 V/cm) 18 min after, respectively.

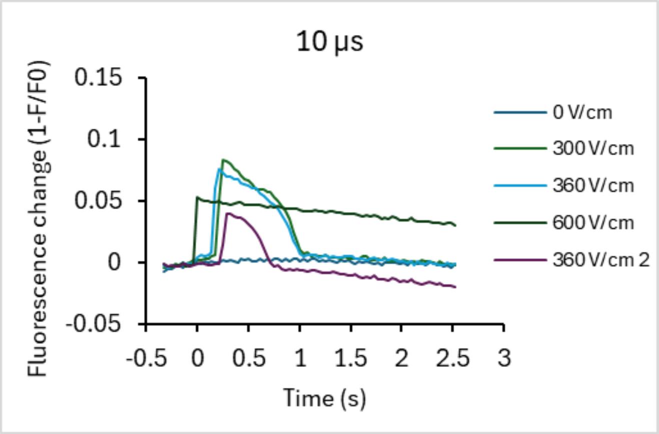

Figure S2: S-HEK cells are still able to trigger action potential (AP) after the entire pulse sequence is completed. Results from a representative experiment. S-HEK cells were exposed to a sequence of 10 µs electric pulses with increasing electric field (from 240 V/cm to 700 V/cm) every 2 min. The last few highest E provoked sustained depolarization. 5 min after the last pulse in the sequence was applied, an additional electric pulse of lower E (10 µs, 360 V/cm, marked in purple as 360 V/cm 2) was applied, resulting in an AP. The lower AP amplitude is likely related to lower potentiometric dye sensitivity due to gradual internalization of the dye from the plasma membranes into cells over time but may also be a consequence of cumulative effects of electroporation. Such experiments were performed 12 times and the ability of cells to generate an AP after the pulse sequence was always confirmed.

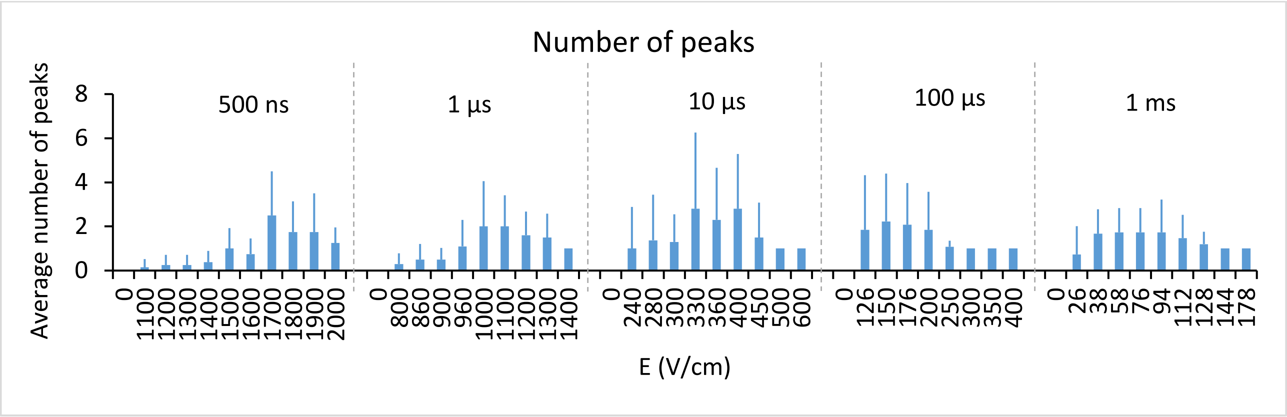

Figure S3: Average number of transmembrane voltage (TMV) peaks for each electric pulse duration and increasing electric field E. The results are presented as mean + SD. Number of experiments (N): 500 ns: 8, 1 µs: 10, 10 µs: 10, 100 µs: 13, 1 ms: 15.

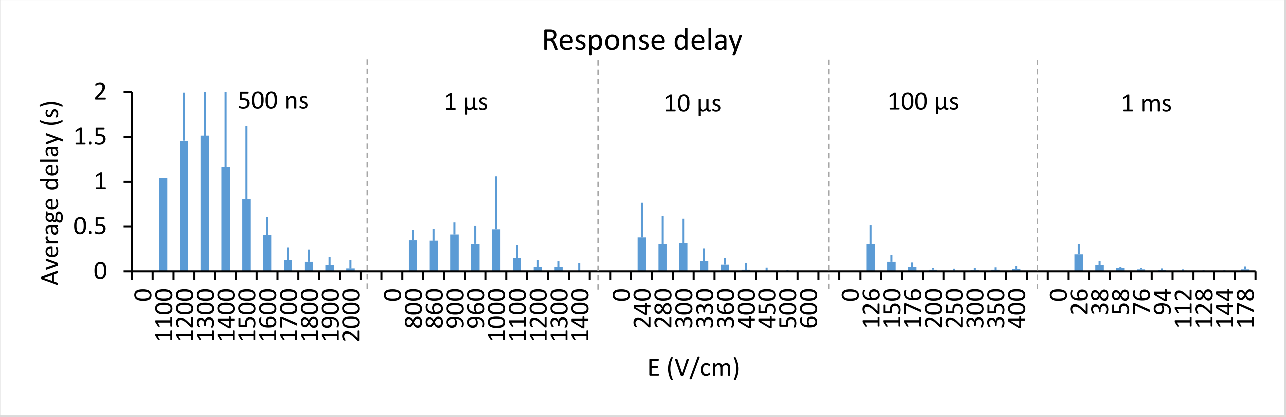

Figure S4: Average TMV peak delay vs. the applied electric field E for each electric pulse duration. The results are presented as mean + SD. Number of experiments (N): 500 ns: 8, 1 µs: 10, 10 µs: 10, 100 µs: 13, 1 ms: 15.
