# Supplementary material for: Excitation and electroporation in genetically engineered excitable S-HEK cells exposed to electric pulses of different durations
Source: Sci Rep. 2025 Jul 2;15:23451. doi: 10.1038/s41598-025-06989-5 (PMC12223188; doi:10.1038/s41598-025-06989-5)
Supplement: Supplementary file 2 — Supplementary Information 2. [file 41598_2025_6989_MOESM2_ESM.pdf]

Supplementary information 2

Excitation and electroporation in genetically engineered excitable S-HEK cells exposed to electric pulses of different durations

Tina Batista Napotnik, Tina Cimperman, Lea Rems

University of Ljubljana, Faculty of Electrical Engineering, Tržaška cesta 25, 1000 Ljubljana, Slovenia

Classification of TMV responses

We divided the TMV responses using a simple rule-based classification. We primarily aimed to classify the responses based on their duration, as we know from our previous study that action potential (AP) prolongation and sustained depolarization indicate electroporation.[1] Based on preliminary analysis, we found that the time from the first peak to 50% and 75% (t_{50}, and t_{75}, respectively) and their ratio t_{75} / t_{50} are good descriptors of the response shape. Fig. S3 shows t_{50} plotted against t_{75} / t_{50} for all responses from all experiments presented in the paper. Grey circles show all responses with a single peak, whereas yellow circles show all responses with multiple peaks. Responses that did not reach t_{75} within the observation time (~2.5 s) are shown with black circles at arbitrary position of t_{75}/t_{50} = 7. Responses that did not reach t_{75} and t_{50} within the observation time are not presented in the plot. The diamonds show the first and second responses obtained with application of 1 ms pulse above the threshold electric field. We consider these responses as normal APs triggered by classical electrostimulation. All these responses had $t_{50} \leq$ 450 ms and t_{75}/t_{50} < 1.8, except for one that had an unusually long t_{50}. For 1 ms pulse there were 3 samples that showed a small peak at the threshold electric field strength followed by a typical AP response at subsequent E. Those small peaks are not presented with diamonds.

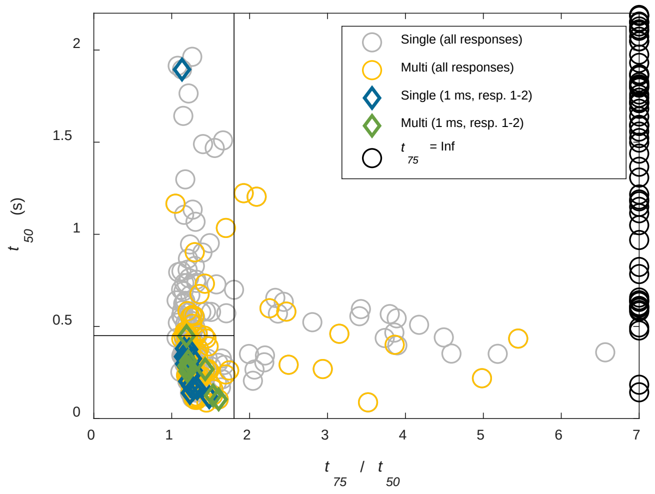

Figure S3: Graph showing t_{50} plotted against t_{75} / t_{50} for all responses from all experiments. Grey and yellow circles show all responses with a single and multiple peak, respectively. Note that there is no visual separation between responses with single and multiple peaks with respect to t_{50} and t_{75} / t_{50}. Responses that did not reach t_{75} within the observation time (~2.5 s) are shown with black circles at arbitrary position of t_{75}/t_{50} = 7. The horizontal line marks t_{50} = 450 ms, the vertical line marks t_{75}/t_{50} = 1.8. We consider that $t_{50} \leq$ 450 ms and $t_{75}/t_{50} \leq$ 1.8 normal APs triggered by classical electrostimulation, since practically all first and second responses obtained with 1 ms pulse above the threshold electric field strength meet these conditions (diamonds).

Based on this analysis we used the following rules to divide the responses:

- If the response has no detected peaks (N_{peaks} = 0), then class = "**No response**".
- If the response has a single peak (N_{peaks} = 1), its normalized amplitude (peak height normalized to the maximal response of the sample) is <0.5 and this is the first response above the threshold electric field, then class = "**Small response**".
- If the response has a single peak (N_{peaks} = 1) and $t_{50} \leq 450$ ms and $t_{75}/t_{50} \leq 1.8$, then class = "**Single AP**".
- If the response has multiple peaks (N_{peaks} > 1) and $t_{50} \leq 450$ ms and $t_{75}/t_{50} \leq 1.8$, then class = "**Multi AP**".
- If the response has a single peak (N_{peaks} = 1) and t_{75} exists, then class = "**Single AP prolonged**".
- If the response has multiple peaks (N_{peaks} > 1) and t_{75} exists, then class = "**Multi AP prolonged**".
- If t_{75} does not exist, class = "**Depolarization**".

All responses corresponding to a given class are presented in Fig. S4.

We stress that there is no sharp transition between an AP and a prolonged AP. Indeed, computational simulations demonstrate that an increasing ionic leak due to electroporation progressively prolongs AP until reaching a sustained depolarization.[1] We introduced a sharp separation at $t_{50} \leq 450$ ms to be able to determine the onset of AP prolongation. While the separation at $t_{50} \leq 450$ ms is somewhat arbitrary, the classification based on this separation captured well our experimental observations.

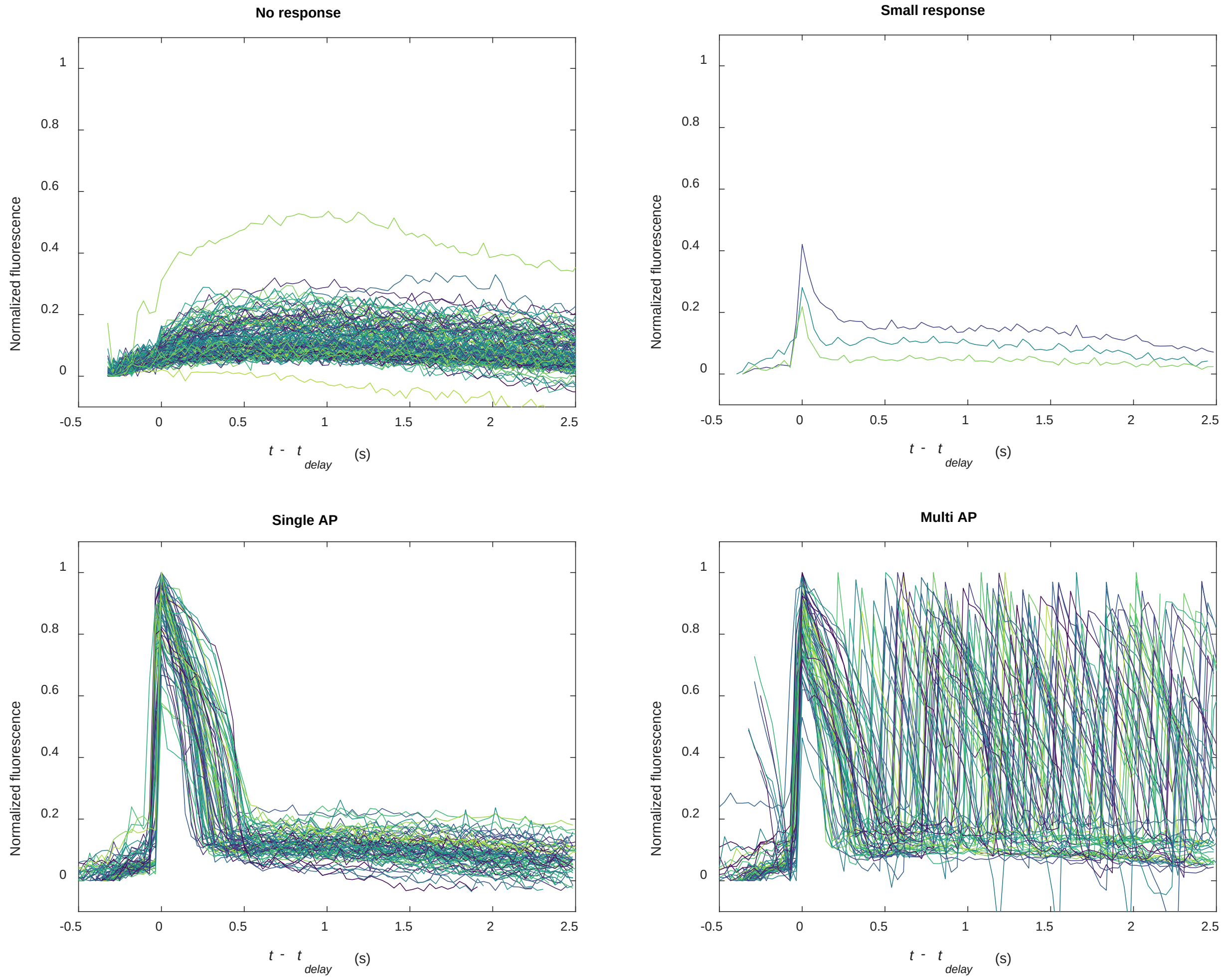

Figure S4: Division of TMV responses to one of seven classes using rule-based classification, as described in the text. For clear comparison between response shapes, each response is normalized to the maximal response of the sample and shifted in time, such that the first peak appears at t = 0 s.

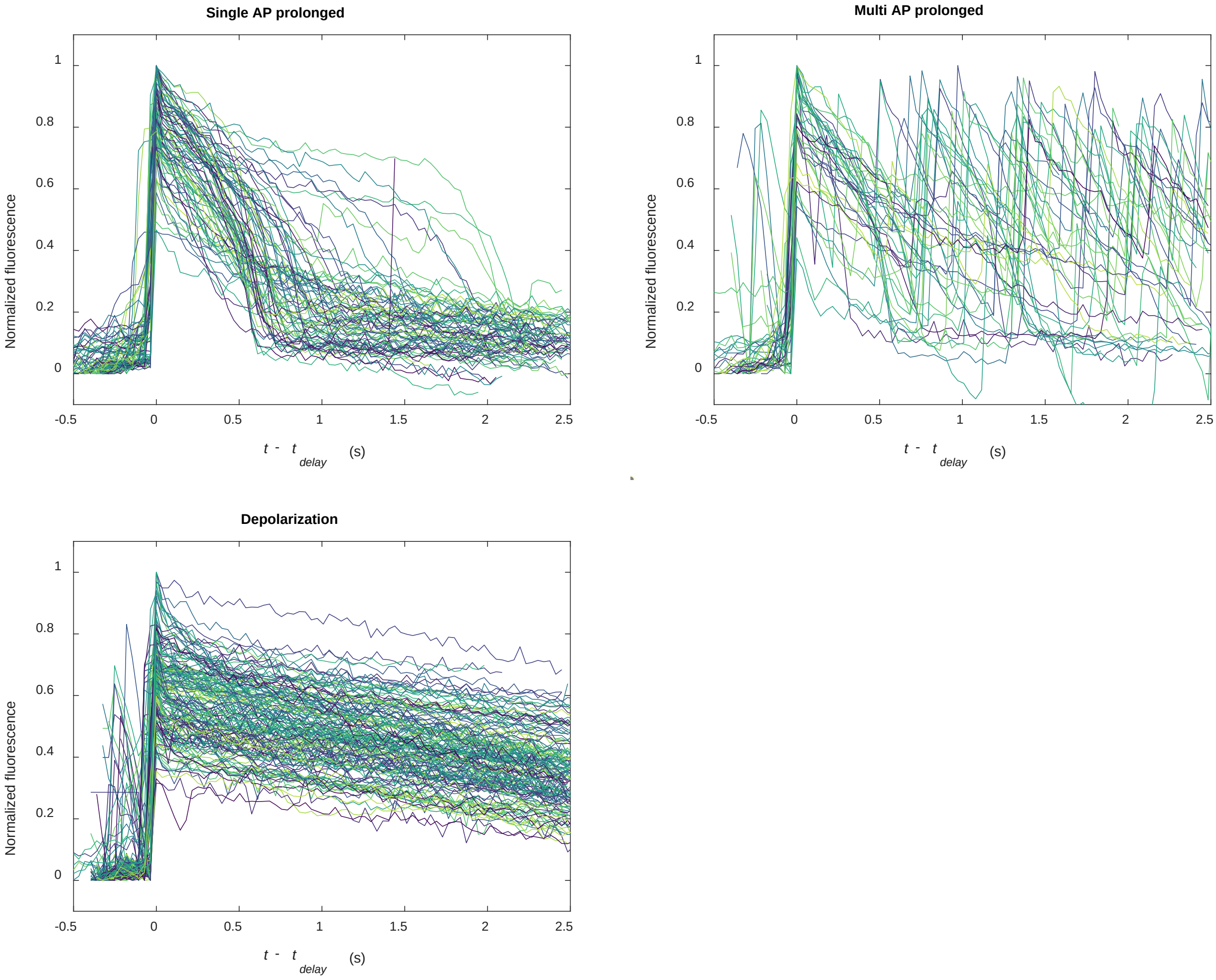

Figure S4 - continued.

Reference

1. Batista Napotnik, T. *et al*. Genetically engineered HEK cells as a valuable tool for studying electroporation in excitable cells. *Sci Rep* **14**, 720 (2024).
